# A High-Reliability RF MEMS Metal-Contact Switch Based on Al-Sc Alloy

**DOI:** 10.3390/mi14061098

**Published:** 2023-05-23

**Authors:** Zhongxuan Hou, Yongkang Zhang, Chaowei Si, Guowei Han, Yongmei Zhao, Xiaorui Lu, Jiahui Liu, Jin Ning, Tongbo Wei

**Affiliations:** 1 Engineering Research Center for Semiconductor Integrated Technology, Institute of Semiconductors, Chinese Academy of Sciences, Beijing 100083, China; houzhongxuan@semi.ac.cn (Z.H.); zhangyongkang@semi.ac.cn (Y.Z.); schw@semi.ac.cn (C.S.); hangw1984@semi.ac.cn (G.H.); ymzhao@semi.ac.cn (Y.Z.); luxiaorui@semi.ac.cn (X.L.); liujiahui@semi.ac.cn (J.L.); 2 School of Electronic, Electrical and Communication Engineering, University of Chinese Academy of Sciences, Beijing 100049, China; 3 Center of Materials Science and Optoelectronics Engineering, University of Chinese Academy of Sciences, Beijing 100049, China; 4 State Key Laboratory of Transducer Technology, Chinese Academy of Sciences, Beijing 100083, China; 5 Research and Development Center for Semiconductor Lighting Technology, Institute of Semiconductors, Chinese Academy of Sciences, Beijing 100083, China

**Keywords:** RF MEMS, switch, Al-Sc alloy, S-parameters

## Abstract

This paper presents a new metal-contact RF MEMS switch based on an Al-Sc alloy. The use of an Al-Sc alloy is intended to replace the traditional Au-Au contact, which can greatly improve the hardness of the contact, and thus improve the reliability of the switch. The multi-layer stack structure is adopted to achieve the low switch line resistance and hard contact surface. The polyimide sacrificial layer process is developed and optimized, and the RF switches are fabricated and tested for pull-in voltage, S-parameters, and switching time. The switch shows high isolation of more than 24 dB and a low insertion loss of less than 0.9 dB in the frequency range of 0.1–6 GHz.

## 1. Introduction

In recent years, microelectromechanical systems (MEMSs) have received increasing attention. Radio frequency MEMS (RF MEMS) refers to manufacturing RF devices using MEMS technology to improve the performance of RF systems. RF MEMS switches are the basic components of RF MEMS, which are superior to traditional mechanical and solid-state switches, including field-effect transistor (FET) and PIN diode semiconductor switches. They have the advantages of low insertion loss, high isolation, low power consumption, and high linearity [1,2,3]. In particular, RF MEMS switches can exhibit high performance from DC, up to tens of gigahertz, and have good application prospects in various RF fields, including phase shifters, reconfigurable filters and amplifiers, phased array radar, and so on [4,5].

RF MEMS switches are generally classified according to the contact method and can be divided into the following two categories: resistive switches (metal–metal) and capacitive switches (metal–insulator–metal). For resistive switches, direct contact with metals is used to form signal pathways. In the higher frequency range of microwave transmission, the metal contact is prone to parasitic effects. Therefore, resistive switches are generally used in the low-frequency range. Metal-contact MEMS switches are typically based on a cantilever beam, air bridge, or membrane actuated by a micromechanical actuator to establish and break metal–metal contact.

The performance of metal-contact RF MEMS switches is closely related to the contacts, as it affects many key parameters that determine switch performance, such as switch resistance and insertion loss, power-handling, and reliability [6,7]. Therefore, it is significant to select the appropriate contact materials to improve contact performance. Traditionally, gold has been used as a contact material due to its low volume resistivity, which enables low contact resistance even under small contact forces, and its high compatibility with MEMS manufacturing methods. However, soft contact materials, such as gold, also generate large contact adhesion and are susceptible to contact wear and contact-surface degradation (such as arcing), thereby affecting contact performance [8,9]. Compared to Au, hard contact materials, such as refractory metals, have been actively developed to increase contact reliability, which is considered the most critical issue in contact-based devices. Radant MEMS, Omron, and University of California San Diego (UCSD) switches are typical examples of devices that use hard contact materials to achieve high contact durability, using large actuators with high contact and restoring forces. Radant connects multiple switching devices in parallel, which can achieve low contact resistance, while exhibiting high reliability under the hot-switching condition [10]. Omron uses single crystal silicon actuators to address the potential changes in contact and restoring forces that large area switches may experience due to stress gradients [11]. UCSD uses tethers to make the switch less sensitive to stress, and uses Au-Ru contacts to improve the reliability of the switch [12]. However, the high hardness, large elastic modulus, and high resistivity of hard contact materials also make it difficult to obtain low contact resistance below 2 Ω, which can be easily obtained for soft contact materials, such as gold. Additionally, it should be noted that these harder metals have analytical activity and often form a friction polymer layer on the contact surface, which may lead to unstable contact resistance of the device [13]. So far, typical hard metal materials (such as Ru, Rh, Pt, and Ir) and their alloys for switch contact have been studied, but all of these materials have high contact resistance and are not a perfect substitute for gold. Therefore, it is essential to find a new contact material for RF MEMS switches to improve the contact performance [14,15,16].

In order to improve the reliability and power-handling ability of metal-contact switches, a multi-contact switch structure based on an aluminum–scandium (Al-Sc) alloy is proposed in this paper. Specifically, we present a theoretical analysis of the performance and design trade-offs of the proposed reliability improvement methodology for RF MEMS switches, showing that the switch can achieve good RF performance. In addition, the polyimide sacrificial layer process matching the Al-Sc alloy is introduced and optimized.

## 2. Switch Design Aspects

**Figure 1:**
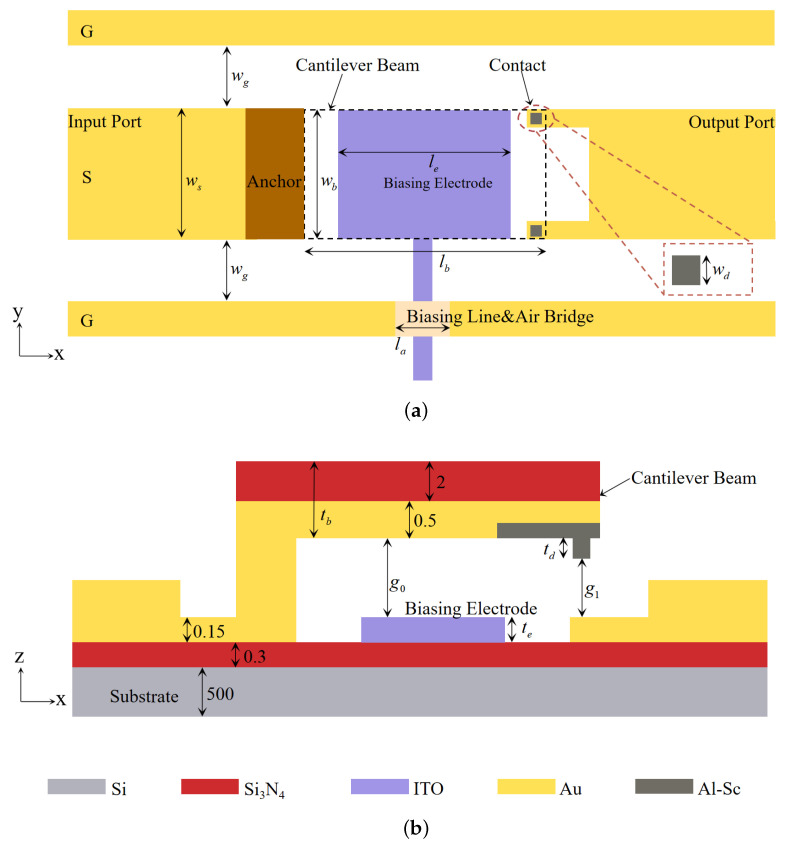
Dimensions of the proposed switch. (**a**) Top view. (**b**) Side view (all dimensions are in μm).

Figure 1 shows the top and side views of the proposed switch, and the critical dimensions are shown in Table 1. The dimension of the cantilever beam is 220 × 100 × 2.5 μm${}^{3}$. The thickness of the contact dimples is 0.5 μm, while the gap between the contacts and the bottom signal line is 2 μm. The gap and contact thickness configuration are set to reduce the pull-in instability. The nominal dimensions of Coplanar Waveguide (CPW) are 100 μm signal-line width and 60 μm gap to the ground lines, resulting in a characteristic impedance of 49.95 Ω on a 500 μm thick high-resistivity substrate. A high-resistive biasing line is used to reduce DC and RF coupling. An air bridge is formed on top of the biasing line to let the biasing line pass through the ground plane. There is no dielectric layer within the DC field to reduce the charging problem. Roman Stefanini et al. designed a miniature RF MEMS metal-contact switch with a small area and low contact and restoring forces [17]. As the size of its cantilever beam is only 25 × 24 × 1.6 μm${}^{3}$, Roman’s switch has a lower sensitivity to stress effects, while resulting in a higher contact resistance. Compared to their switch, the RF switch in this paper has a larger cantilever beam and biasing electrode, providing higher contact and restoring forces, which is significant for a hard-metal-contact switch. Yuhao Liu et al. proposed a large-area switch based on their dual-contact concept [18]. The purpose is to achieve both low contact resistance and high contact force under the hot-switching condition. In Yuhao’s design, the gap between the cantilever beam and the bottom electrode is only 0.9 μm. Compared to their switch, the gap in our switch is 2.5 μm, which can achieve greater off-state isolation and provide greater stress tolerance, allowing the switch to function normally under minor warping or bending conditions.

Two parallel series contacts are placed at the end of the cantilever beam and can be connected to the CPW signal line. In one switching cycle, the switch is initially in the off state. When driving the cantilever beam through the biasing electrode, the top of the contacts touches the signal line. Similar to zipping actuation, the cantilever beam is further deflected downwards with the increase in the driving voltage, resulting in the switch operating in the on state and exhibiting low loss. When the driving voltage decreases, the cantilever beam rebounds under the restoring force, breaking the contact, and the switch returns to the off state and exhibits high isolation. Under the normal operating condition, the cantilever beam does not contact the biasing electrode, so there is no need to set stop dimples below the beam.

The switch cantilever must have sufficient thickness to avoid fracturing during the switch operation. We propose to use a composite structure composed of 500 nm-thick gold and 2 μm-thick Si${}_{3}$N${}_{4}$. The pull-in voltage *V${}_{p}$* can be approximated by [6]

(1) $$V_{p}=\sqrt{\frac{8k}{27\varepsilon_{0}A}g_{0}^{3}}$$

where ε${}_{0}$ is the air dielectric constant, *A* is the area directly opposite the cantilever beam and the driving electrode, *g${}_{0}$* is the distance between the cantilever beam and the driving electrode, and *k* is the equivalent elastic coefficient of the cantilever beam. When calculating the equivalent spring constant of the composite beam, the thickness and Young’s modulus of each layer should be taken into account, which can be approximately given by

(2) $$k=\frac{E_{Au}t_{Au}+E_{{Si}_{3}N_{4}}t_{{Si}_{3}N_{4}}}{t_{b}}\frac{w_{b}}{4}{\left(\frac{t_{b}}{l_{b}}\right)}^{3}$$

where *E${}_{Au}$* and *E${}_{{Si}_{3}N_{4}}$* are the Young’s modulus of Au and Si${}_{3}$N${}_{4}$, *t${}_{Au}$* and *t${}_{{Si}_{3}N_{4}}$* are the thickness of the Au and Si${}_{3}$N${}_{4}$ layers, respectively. *t${}_{b}$* is the thickness, *l${}_{b}$* is the length, and *w${}_{b}$* is the width of the beam.

**Figure 2:**
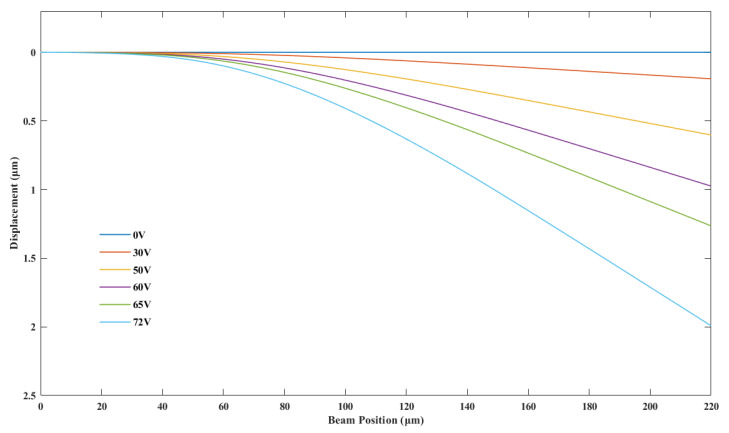
Schematic diagram of simulated cantilever beam displacement changing with driving voltage.

**Figure 3:**
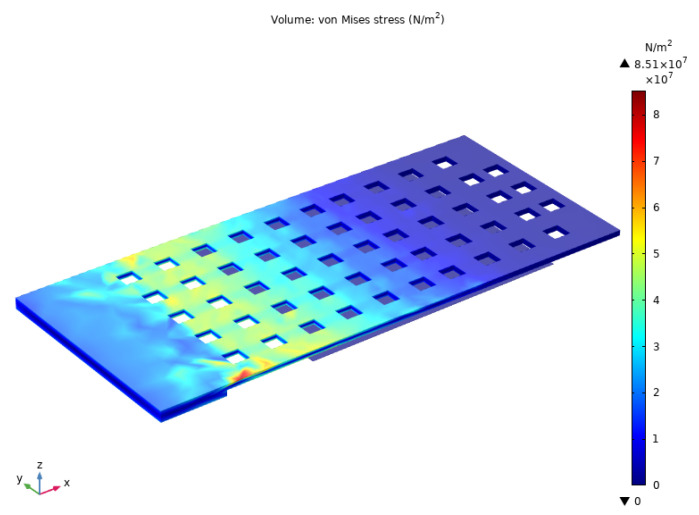
Simulated von Mises stress under 72 V actuation voltage.

The switch cantilever beam has potentially very high residual stress, which is caused by the Plasma Enhanced Chemical Vapor Deposition (PECVD) and sputtering processes of different layers, as in the case of our switch. The high residual stress in the film can lead to adverse effects, such as high pull-in voltage, warping or even fracturing of the film [19]. Small holes (8 × 8 μm${}^{2}$) are defined on the switch cantilever beam, which are beneficial for releasing residual stress. In addition, these release holes can help to release the sacrificial layer more efficiently and completely. Reasonable control of the condition parameters of PECVD Si${}_{3}$N${}_{4}$ can make the Si${}_{3}$N${}_{4}$ film compensate for the compressive stress gradient of the metal layer, making the released cantilever beam flatter. The mechanical behavior of the switch is simulated in finite element analysis software COMSOL Multiphysics (version 5.6, COMSOL, Stockholm, Sweden ) (edition, company, city, state if USA, country). Figure 2 shows the schematic diagram of simulated cantilever beam displacement changing with driving voltage. With the increase in the driving voltage, the cantilever beam displacement increases correspondingly. The displacement of contact dimples can be characterized by the variation of displacement at the beam position of 220 μm. As the gap between the cantilever and the electrode is 2.5 μm and the thickness of the contact dimples is 0.5 μm, the displacement of the contact dimples should be 2 μm when the switch achieves contact between the contact dimples and the signal line. Therefore, the pull-in voltage is considered to be 72 V as is shown in Figure 2. Figure 3 shows the simulated von Mises stress distribution under 72 V actuation voltage. The maximum stress value is 85.1 MPa, which occurs around the release hole on the side adjacent to the switch anchor point.

The most significant advantage of the proposed switch is the use of an Al-Sc alloy as the contact dimples, improving the reliability of the switch. Some studies have shown that adding an appropriate amount of scandium into aluminum can refine the grain size and significantly improve the hardness of the alloy [20]. The Al-Sc alloy also has the advantages of high temperature resistance and corrosion resistance, and has received widespread attention in recent years [21]. Compared to pure gold, the Al-Sc alloy is a harder material with better resistance stability, which can reduce adhesion and contact wear during contact, and can reduce the corrosion caused per arcing operation. However, a much stronger actuator is required to achieve a stable low contact resistance. For a metal-contact RF MEMS switch, the contact resistance can be expressed accurately as [7]

(3) $$R_{c}=R_{a}+Z=\frac{\rho }{2a}+R_{f}$$

(4) $$a=\sqrt{\frac{F_{c}}{\pi H}}$$

where ρ is the resistivity of the contact material, *a* is the equivalent radius of the contact point, and $R_{f}$ is the additional resistance due to resistance contamination. $F_{c}$ is the contact force and *H* is the hardness of the contact material. For a typical RF MEMS switch with 300 μN–1 mN contact force, the contact resistance of which when using the Al-Sc alloy as the contact dimples is about six times higher than that of the switch using the more unstable gold. In addition, the main material of the switch cantilever beam in this article is Au, which has a lower resistivity than the Al-Sc alloy, causing the switch movable beam to have a resistance close to that of pure gold. With the advantage of the hard contact surface, the switch also has relatively low line resistance. It should be noted that the scandium content of the Al-Sc alloy used in this article is 0.6% wt. An Al-Sc alloy film with a thickness of 500 nm is sputtered on a glass substrate for measurements. The hardness value analysis of Al-Sc alloy thin films is obtained by a nanoindentation test, which is a widely used method for determining the hardness and Young’s modulus of thin films [22]. Using Nanoindenter XP (MTS System Corporation, Eden Prairie, Minnesota, United States (city, state if USA, country)), five indentations were measured on each sample. The measurement results show the hardness of the Al-Sc alloy film *H${}_{Al-Sc}$* = 5.1 GPa, and the hardness of the gold film *H${}_{Au}$* = 1.6 Gpa. The resistivity of the Al-Sc alloy thin film is obtained using a four-probe test method, with a value of ρ${}_{Al-Sc}$ = 8.17 × 10${}^{-8}$ Ω ·m. The resistivity of gold thin film (ρ${}_{Au}$) is 2.40 × 10${}^{-8}$ Ω ·m.

**Figure 4:**
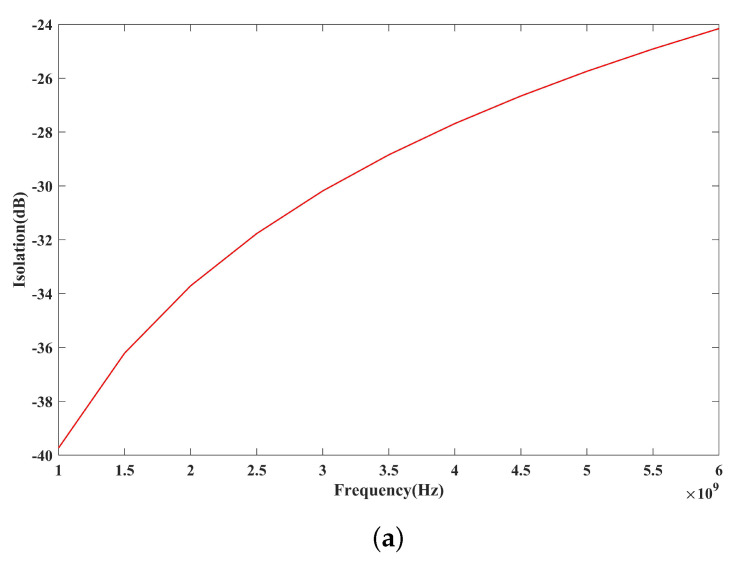

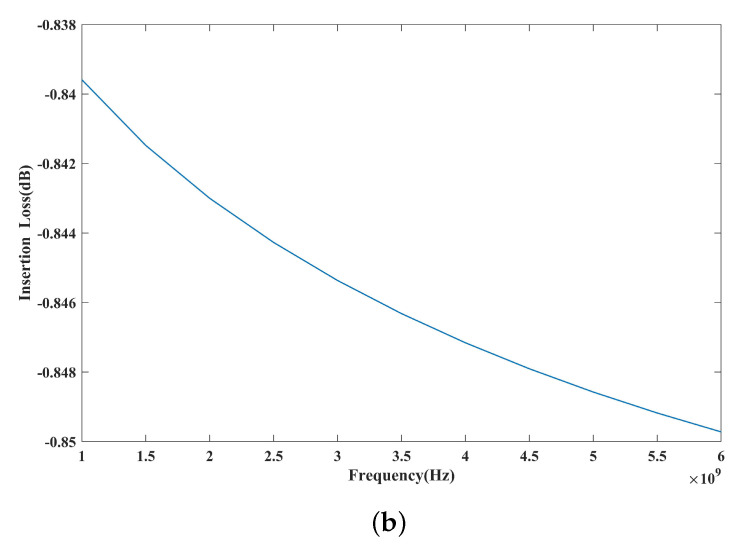
Simulated RF performance results of the proposed RF MEMS switch. (**a**) Off state. (**b**) On state.

Figure 4 shows the simulated RF performance results of the proposed switch in various states. Simulations are carried out in COMSOL Multiphysics. For metal-contact switches, the increase in contact resistance will directly lead to the increase in insertion loss. Therefore, it is necessary to consider the influence of the contact resistance when simulating the insertion loss under bias. In this paper, we conduct geometric modeling according to the dimensions of CPW and the cantilever beam in our design. It should be noted that during modeling, the working state of the switch under pull-in voltage is equivalent to achieving good contact between the contact and signal lines with the contact resistance of 8 Ω, which is calculated by Equations (3) and (4). The simulated off-state isolation is 39.7 dB at 1 GHz and 24.1 dB at 6 GHz before biasing. The on-state insertion loss is 0.839 dB at 1 GHz and 0.849 dB at 6 GHz after actuation.

## 3. Fabrication

**Figure 5:**
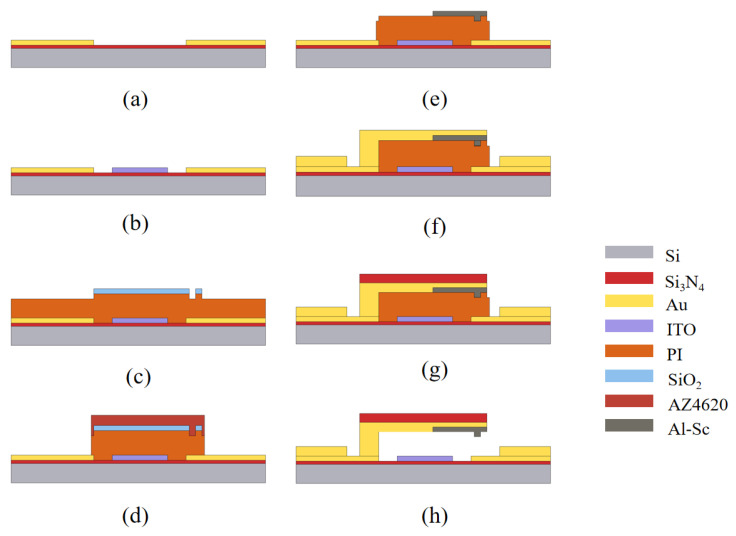
Fabrication process of the switch. (**a**) Patterning of bottom electrode; (**b**) Patterning of ITO DC biasing line; (**c**) Formation of cantilever contact mold; (**d**) Patterning of PI sacrificial layer; (**e**) Patterning of the Al-Sc alloy; (**f**) Formation of the cantilever beam; (**g**) Thickening of cantilever beam; (**h**) Removal of the PI sacrificial layer.

**Figure 6:**
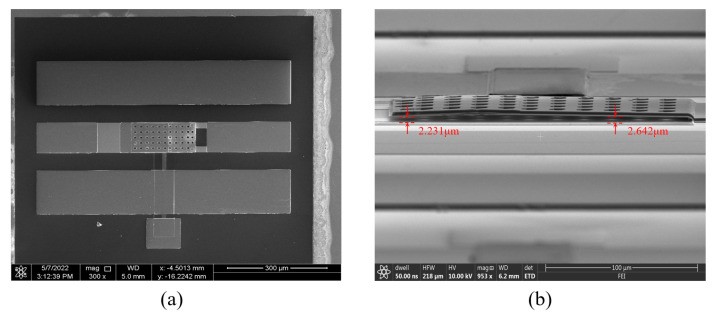
SEM images of the fabricated RF MEMS switch. (**a**) Top view. (**b**) Side view.

Figure 5 shows the key process of switch manufacturing. RF MEMS switches are fabricated on a 500 μm high resistivity silicon wafer, which is covered by a 300 nm-thick Si${}_{3}$N${}_{4}$ layer. The 150 nm-thick gold with a 50 nm Ti adhesive layer is first patterned by lift-off (Figure 5a). Next, a second lift-off is used to pattern a 150 nm-thick high-resistivity ITO DC biasing line (Figure 5b). Then, 2.5 μm-thick polyimide (PI) is rotationally coated to form a sacrificial layer. It is also worth mentioning that the patterning of PI includes two steps. The first step is to use silicon dioxide as a mask and form a 500 nm-thick cantilever contact mold by inductively coupled plasma (ICP) etching (Figure 5c). In the second step, AZ4620 positive photoresist (Merck KGaA, Darmstadt, Germany) (company, city, state if USA, country) is used as a mask, and the PI is patterned by ICP etching (Figure 5d). Then, the 500 nm thick Al-Sc alloy is patterned by lift-off (Figure 5e). In this step, the cantilever contact dimples are formed in Al-Sc. Ti/Au/Ti with a thickness of 30/500/30 nm is patterned to form the cantilever beam and thicken the bottom CPW structure (Figure 5f). The Si3N4 structural layer with a thickness of 2 μm is deposited through a PECVD system and patterned by ICP. The cantilever beam is a multi-layer structure with a thickness of 2.5 μm (Figure 5g). Finally, oxygen plasma is used to remove the sacrificial layer (Figure 5h). Figure 6 shows SEM images of the fabricated switch. As is shown in Figure 6b, the gap between the fixed end of the cantilever beam and the bottom electrode is 2.642 μm. Compared to the design value of 2.5 μm, the gap is slightly larger, as there is an inevitable deviation for the thickness of PI film during its formation. In addition, the gap between the free end of the cantilever beam and the bottom electrode is 2.231 μm, showing that there is a small degree of bending at the free end of the switch due to the stress during the removal of the sacrificial layer. The decrease in the gap does not have a significant impact on the pull-in voltage.

## 4. Measurement and Discussion

All tests are conducted in an open laboratory environment without temperature measurement or pressure control. The pull-in voltage of the switch is tested using Keysight B1500, which is 75–80 V for most devices. The test result is larger than the simulated 72 V and fluctuates within a certain range, mainly due to the slight upwarping of the switch cantilever beam caused by the residual stress in the process of removing the sacrificial layer.

**Figure 7:**
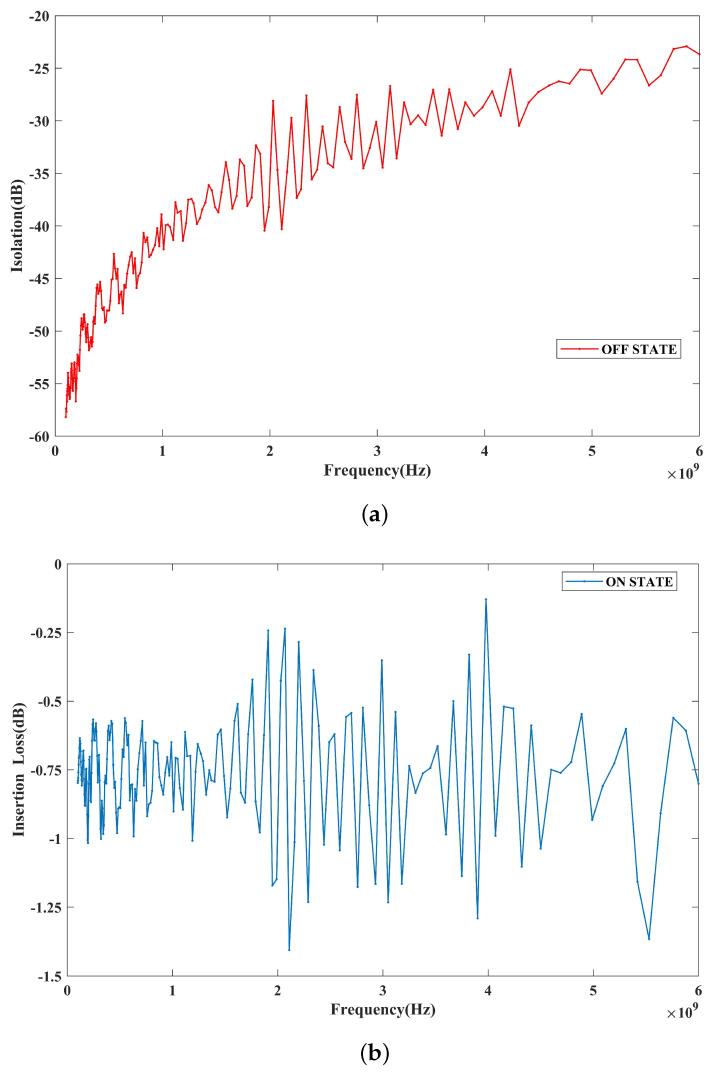
Measured S−parameters. (**a**) Off state. (**b**) On state.

The small-signal RF performance results of the fabricated switches are measured using an R&S ZVL network analyzer with a ground–signal–ground (G-S-G) microwave probe. Figure 7 shows the test results for the S-parameters. The off-state isolation of the switch is shown in Figure 7a. The isolation of the switch is 58 dB at 0.1 GHz and 24 dB at 6 GHz. The measured isolation results agree well with the finite element simulation results. The on-state insertion loss of the switch is shown in Figure 7b. To achieve good contact, the switch is biased at 83 V and has an insertion loss of less than 0.9 dB in the range of 0.1–6 GHz. The measured insertion loss is larger than that obtained from the simulation and fluctuates within a small range. This is because the contact resistance value during switch operation is not stable, and there is a slight change. For Au-Au contact switches, there are numerical publications and commercial products. The switch designed by E. Jouin et al. obtains the on state by applying a 75 V bias voltage, with an insertion loss of 0.6 dB at 6 GHz [23]. In comparison, the insertion loss of our switch increases at the same frequency, which is mainly caused by the larger contact resistance. The tested insertion loss gives a resistance of 20 Ω, including the switch contact resistance and the loss of the entire circuit. By testing the same switch in the on state, we find that the total resistance of the RF line is 12 Ω. Therefore, a contact resistance of 8 Ω is extracted. Considering that many research works have shown that some hard metal contacts, such as Pt-Pt, have a contact resistance of over 100 Ω [13,24], our switch contact resistance is acceptable and can meet the needs of practical applications.

**Figure 8:**
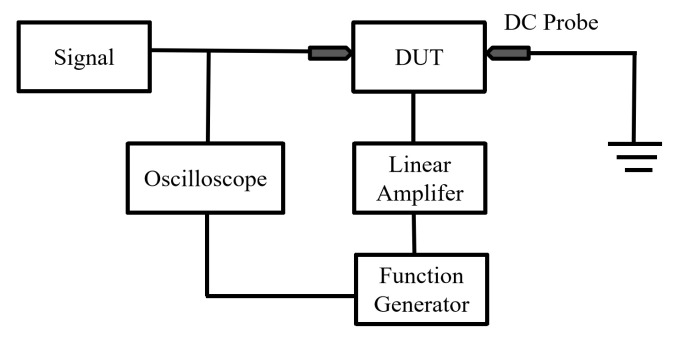
Test setup schematics for switching time measurement.

**Figure 9:**
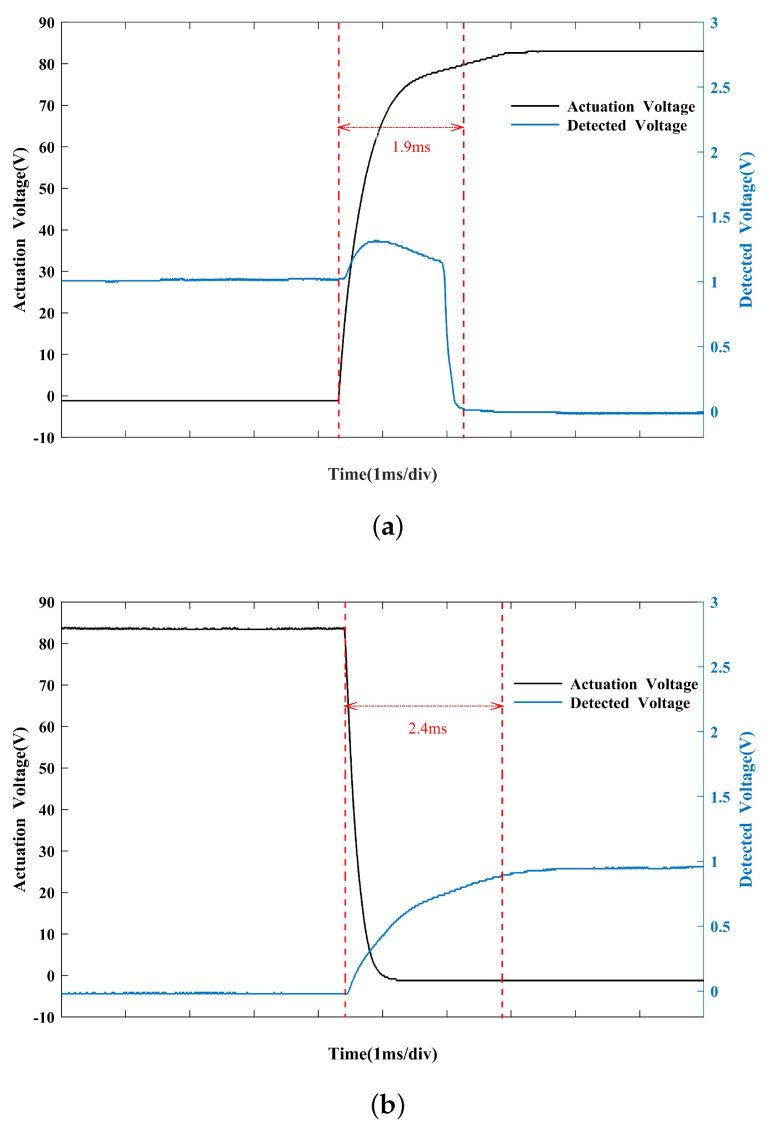
(**a**) Switching−on time, (**b**) switching−off time.

The test device for switching time is shown in Figure 8. The device is driven by a bias signal generated by a function generator and amplified by a linear amplifier. The resulting drive signal is a 100 Hz square wave of 0–83 V, with a duty cycle of 50%. The oscilloscope detects the signals at the switch(DUT) input and bias ends to determine the switching time. As shown in Figure 9, the switching-on time is 1.9 ms and the switching-off time is 2.4 ms.

## 5. Conclusions

In this paper, the design and experimental validation of a new type of metal-contact RF MEMS switch with high reliability is presented and discussed. The switch design is accomplished by contacting an Al-Sc alloy with gold. The contact hardness is more than three times larger than that of pure gold. In addition, a multi-layer stack structure is adopted to combine the advantages of the low resistivity of gold and the hard contact surface. Moreover, a new method to form a polyimide sacrificial layer is proposed for the multi-layer stack structure. The new RF MEMS switch is tested in an atmospheric environment. In our test, the switch exhibits a high isolation of more than 24 dB and a low insertion loss of less than 0.9 dB in the frequency range of 0.1–6 GHz. Compared to traditional switches that use hard metal contacts instead of Au-Au contacts, it has lower contact resistance and better RF performance results.

## Figures and Tables

**Table 1:** Geometry parameter of the switch.

Geometry Parameter	Symbol	Value (μm)
CPW line width	*w* ${}_{s}$	100
CPW line gap	*w* ${}_{g}$	60
Beam width	*w* ${}_{b}$	100
Dimple width	*w* ${}_{d}$	10
Beam length	*l* ${}_{b}$	200
Biasing electrode length	*l* ${}_{e}$	120
Air bridge width	*l* ${}_{a}$	60
Beam thickness	*t* ${}_{b}$	2.5
Dimple thickness	*t* ${}_{d}$	0.5
Biasing electrode thickness	*t* ${}_{e}$	0.15
Cantilever to electrode gap	*g* ${}_{0}$	2.5
Dimple to contact gap	*g* ${}_{1}$	2

## Data Availability

Not applicable.
